# Impact of 1.5 ^o^C and 2 ^o^C global warming scenarios on malaria transmission in East Africa

**DOI:** 10.12688/aasopenres.13074.3

**Published:** 2021-03-15

**Authors:** Obed Matundura Ogega, Moses Alobo

**Affiliations:** 1 Programmes, The African Academy of Sciences, Nairobi, Kenya; 2 School of Environmental Studies, Kenyatta University, Nairobi, Kenya

**Keywords:** SR1.5, CORDEX, malaria, RCP 8.5, global warming, mosquito vectors

## Abstract

**Background:** Malaria remains a global challenge with approximately 228 million cases and 405,000 malaria-related deaths reported in 2018 alone; 93% of which were in sub-Saharan Africa. Aware of the critical role than environmental factors play in malaria transmission, this study aimed at assessing the relationship between precipitation, temperature, and clinical malaria cases in East Africa and how the relationship may change under 1.5 ^o^C and 2.0 ^o^C global warming levels (hereinafter GWL1.5 and GWL2.0, respectively).

**Methods:** A correlation analysis was done to establish the current relationship between annual precipitation, mean temperature, and clinical malaria cases. Differences between annual precipitation and mean temperature value projections for periods 2008-2037 and 2023-2052 (corresponding to GWL1.5 and GWL2.0, respectively), relative to the control period (1977-2005), were computed to determine how malaria transmission may change under the two global warming scenarios.

**Results**: A predominantly positive/negative correlation between clinical malaria cases and temperature/precipitation was observed. Relative to the control period, no major significant changes in precipitation were shown in both warming scenarios. However, an increase in temperature of between 0.5 ^o^C and 1.5 ^o^C and 1.0 ^o^C to 2.0 ^o^C under GWL1.5 and GWL2.0, respectively, was recorded. Hence, more areas in East Africa are likely to be exposed to temperature thresholds favourable for increased malaria vector abundance and, hence, potentially intensify malaria transmission in the region.

**Conclusions**: GWL1.5 and GWL2.0 scenarios are likely to intensify malaria transmission in East Africa. Ongoing interventions should, therefore, be intensified to sustain the gains made towards malaria elimination in East Africa in a warming climate.

## Introduction

Malaria is an illness caused by *Plasmodium* parasites that are spread to humans through bites of infected female *Anopheles* mosquitoes, commonly referred to as “malaria vectors”. Of the five parasite species that cause malaria in humans, *P. falciparum and P. vivax* pose the highest threat ( WHO, 2020). According to the World Health Organization (WHO), an estimated 228 million malaria cases and 405,000 malaria-related deaths were reported in 2018, globally. About 93% of the malaria cases and 94% of the malaria-related deaths occurred in sub-Saharan Africa. Uganda, for instance, tops East Africa with the highest number of malaria cases; accounting for 5% of global totals in 2018.

Malaria transmission is affected by, among other things, climatic factors such as temperature, rainfall, and humidity that influence the abundance and survival of mosquitoes ( Caminade *et al*., 2012; Metelmann *et al*., 2019; Nsoesie *et al*., 2016). While efforts are underway towards elimination, malaria remains a big challenge in East Africa ( Bashir *et al*., 2019; Nkumama *et al*., 2017; WHO, 2020). In a special report on global warming of 1.5 °C (hereinafter SR1.5), the Intergovernmental Panel on Climate Change (IPCC; Hoegh-Guldberg *et al*., 2018) highlighted sector-specific risks posed by a global temperature rise of 1.5 °C and beyond. The SR1.5 identifies a knowledge gap in the impacts of global and regional climate change at 1.5 °C on, *inter alia*, public health and infectious diseases, particularly for developing nations. Some work has been done towards understanding the potential impact of global warming in East Africa (e.g. Gudoshava *et al*., 2020; Osima *et al*., 2018). However, no conclusive literature exists on the potential impacts of 1.5 °C and 2 °C global warming levels (hereinafter GWL1.5 and GWL2.0) on health, among other sectors, in East Africa. This study, therefore, aimed at assessing the relationship between precipitation, temperature, and clinical malaria cases in East Africa and how the relationship may change under the GWL1.5 and GWL2.0 scenarios.

## Methodology

### Study area

**Figure 1.**
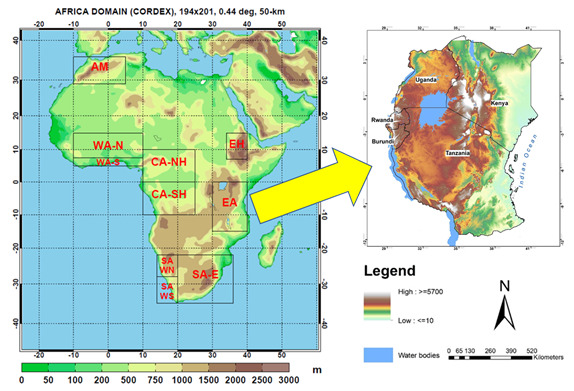
Map of the study domain. Figure is reproduced from Ogega *et al*. (2020) under the terms of the Creative Commons Attribution 4.0 International license (CC-BY 4.0).

The study focuses on the East Africa sub-region (marked EA on Figure 1) of the COordinated regional Downscaling Experiment (CORDEX) Africa domain ( Kim *et al*., 2014). A slight extension of the CORDEX-EA sub-region was done to cover five countries part of the East African Community (EAC) namely Kenya, Uganda, Tanzania, Rwanda, and Burundi ( Figure 1).

### Climate model data

Daily precipitation data (in its native form) from two regional climate models (RCMs) participating in CORDEX-Africa were used. Specifically, the study used four RCM realizations ( Table 1) driven by general circulation models (GCMs) from the 5 ^th^ phase of the Coupled Model Intercomparison Project (CMIP5, Meehl *et al*., 2014), under the representative concentration pathway (RCP) 8.5 ( Moss *et al*., 2010). Here, we chose the RCP 8.5 due to its more realistic representation of global warming scenario considering todays’ global greenhouse gas emission trajectory ( Taylor *et al*., 2012). Additionally, the RCP 8.5 has been widely used in Africa and beyond (e.g. Gudoshava *et al*., 2020; Ogega *et al*., 2020; Yan *et al*., 2019). The four CORDEX-Africa RCM runs have been identified to be among the best in simulating precipitation characteristics over East Africa ( Ogega *et al*., 2020). The RCMs are described in detail in Nikulin *et al*. (2012).

**Table 1.** CORDEX-Africa RCM runs used in the current study, downloaded in April 2020 from the Deutsches Klimarechenzentrum (DKRZ ^1^), for the period 1977–2005 (historical) and 2008–2052 (RCP 8.5).

Institute	RCM	Herein-after	Ensemble	Driving Model
Max Planck Institute (MPI), Germany	REMO2009	REMO2009	r1i1p1	MPI-M-MPI-ESM-LR
Sveriges Meteorologiska och Hydrologiska Institut (SMHI), Sweden	SMHI Rossby Center Regional Atmospheric Model (RCA4)	RCA4	r1i1p1	MPI-M-MPI-ESM-LR
				CNRM-CERFACS-CNRM-CM5
			r2i1p1	MPI-M-MPI-ESM-LR

The terms in the table can be used to search for the required data files

### Observational climate data

The daily Climate Hazards Group InfraRed Precipitation with Station data (CHIRPS) version 2.0 was used as observational precipitation data. CHIRPS data, which have been validated for East Africa ( Dinku *et al*., 2018), incorporate satellite imagery (at 0.05° resolution) with *in-situ* station data resulting in a gridded rainfall time series available from 1981 to near-present ( Funk *et al*., 2015). For mean temperature, the Climatic Research Unit time-series (CRU) dataset were used. CRU data are computed on high-resolution (0.5 by 0.5 degree) grids based on a database of monthly mean temperatures from at least 4,000 weather stations from around the world ( Harris *et al*., 2020).

### Clinical malaria cases data

Data on clinical malaria cases for East Africa were obtained from the Malaria Atlas Project ( Hay & Snow, 2006; Weiss *et al*., 2019). The Malaria Atlas Project (MAP) obtains, curates, and shares a variety of malariometric data including malaria cases reported by surveillance systems, nationally representative cross-sectional surveys of parasite rate, and satellite imagery capturing global environmental conditions that influence malaria transmission. The dataset has been validated (e.g. Nakakana *et al*., 2020) and used widely across the world (e.g. Battle *et al*., 2019; Bhatt *et al*., 2015; Weiss *et al*., 2019).

## Data analysis

Precipitation and temperature have been identified as the most important climatic factors for malaria vectors (e.g. Arab *et al*., 2014; Mohammadkhani *et al*., 2016). In the current study, a review of literature was done to identify precipitation and temperature thresholds within which malaria vectors thrive. The search was done in Scopus and Google Scholar using the following terms: temperature threshold for *Anopheles* mosquitos, precipitation threshold for *Anopheles* mosquitos, and malaria transmission in East Africa. Results of the review were used to analyse historical (2000–2017, due to limited availability of data on clinical malaria cases from MAP) trends in temperature, precipitation, and clinical malaria cases in East Africa. Specifically, standardized anomalies, which remove influences of location and distribution from the data (as in Dabernig *et al*., 2016), were computed to determine the year-to-year variability of incidences. Linearly de-trended precipitation and temperature data were used for correlation analysis with reference to the reported clinical malaria cases in the study domain.

A detailed analysis done by ( Nikulin *et al*. (2018) identified years 2022 and 2037 as mid-years for 30-year periods when GWL1.5 and GWL2.0 were likely to be experienced in Africa, respectively, using an ensemble mean of a subset of GCMs driving the CORDEX-Africa RCM realizations. Our study adapted periods 2008–2037 and 2023–2052 to correspond to GWL1.5 and GWL2.0, respectively. With reference to 1977–2005 as the control period (CTL), we assessed changes in precipitation and temperature by calculating differences between climatological values in GWL1.5 and GWL2.0 and the CTL. A comparison of precipitation and temperature values in the current (CTL), GWL1.5 and GWL2.0 (relative to established thresholds within which malaria vectors thrive) was used to determine the potential impact of 1.5 °C and 2.0 °C GWLs on malaria transmission in East Africa.

### Statistical computations and data visualization

Processing (conversion to common calendar, units, grid, and resolution) and statistical computations (e.g. means, anomalies, standard deviation, summations, and data detrending) of climate (precipitation and temperature) data in NetCDF format was done using the Climate Data Operators (CDO), version 1.9.8 – a command line suite for manipulating and analysing climate data. A description of CDO operators is available from the CDO user guide. Additional computations were done using the R Project for Statistical Computing (R, version 3.6.3). Specifically, the *fields, graphics, and ncdf4* R packages were used to process and compute future changes in precipitation and temperature under the 95% confidence level. Data detrending and correlation analysis were done in R using the *pracma* package and the *cor.test* function, respectively. Spatial data visualization was done using the Grid Analysis and Display System ( GrADS, version 2.2.1.oga.1). Line plots were done in R using the *ggplot2 (version 3.3.0)* package.

Due to resolution differences between model and observations data, the data were processed in their native grids before bi-linearly interpolating them to the RCM grid to facilitate comparison (as in Diaconescu *et al*., 2015). Here, final products (after all the statistical computations) for both observational and model data were remapped into the same grid to facilitate comparison. Remapping was done using the *‘remapbil’* function in the CDO software.

## Results and discussion

### An overview of the relationship between temperature, precipitation, and malaria vectors

*An. gambiae s.s., An. funestus*, and *An. arabiensis* have been identified as the top three potent malaria vectors in sub-Saharan Africa ( Wiebe *et al*., 2017) and, in particular, East Africa (e.g. Dida *et al*., 2018; Karungu *et al*., 2019). Among other climatic factors that influence malaria transmission is temperature – which has been shown to be a useful predictor of incidence (e.g. Pascual *et al*., 2006). Any climate-induced changes in temperature are likely to disproportionately affect malaria control interventions across the world (e.g. Ryan *et al*., 2020; Siraj *et al*., 2014). A study by ( Charlwood, 2017) established that *An. funestus* seemed to be adversely affected by temperatures above 28 °C. Additionally, the wing size of *An. funestus* is said to be highly correlated with temperature and elevation (Spearman test, p<0.001) and minimally affected by rainfall and wind speed ( Ayala *et al*., 2011). Christiansen-Jucht *et al*. (2014) inferred that temperature during larval development and adult maintenance influences the survival of *An. gambiae* s.s. Their study established that temperatures beyond 27 °C significantly influenced the survival of adult *An. gambiae* s.s. by increasing their mortality.

In areas where malaria transmission by *An. funestus* is high, transmissions by *An. gambiae* s.s. and *An. arabiensis* seemed to be higher/lower with precipitation/temperature ( Kelly-Hope *et al*., 2009). Further, a temperature-dependent and stage-structured delayed differential equation developed by Beck-Johnson *et al*. (2013) showed that mosquito population abundance is strongly influenced by the dynamics of juvenile mosquito stages which are temperature-dependent. Their model places a peak in abundance of mosquitoes old enough to transmit malaria at around 25 °C. Generally, studies have shown that significant malaria transmission in Africa occurs in areas with temperature ranging from 18 °C to 28 °C, with 25 °C as the optimum temperature (e.g. Craig *et al.,* 1999; Mordecai *et al.,* 2013). Hence, our study adopted the 18 °C – 28 °C as the temperature range within which significant malaria transmission occurs.

While no distinct annual precipitation thresholds have been established for malaria transmission, precipitation plays a vital role in both mosquito abundance and spatial and temporal malaria transmission. It does so by providing good aquatic environments to host malaria vectors. Indeed, heavy and extreme precipitation events have been associated with higher malaria cases in East Africa (e.g. Brown *et al.,* 1998; Hashizume *et al.,* 2009; Kilian *et al.,* 1999). For instance, a study by ( Gilioli & Mariani (2011) established that a 10 percent increase in precipitation can result in about 6 percent increase in mosquito population in Kenya. Therefore, we assessed the climatology for mean annual precipitation and how it may change under GWL1.5 and GWL2.0. The changes in precipitation patterns will give an indication of the intensity and extent of malaria transmission under the two warming scenarios.

### Trends in temperature, precipitation, and clinical malaria cases in East Africa

**Figure 2.**
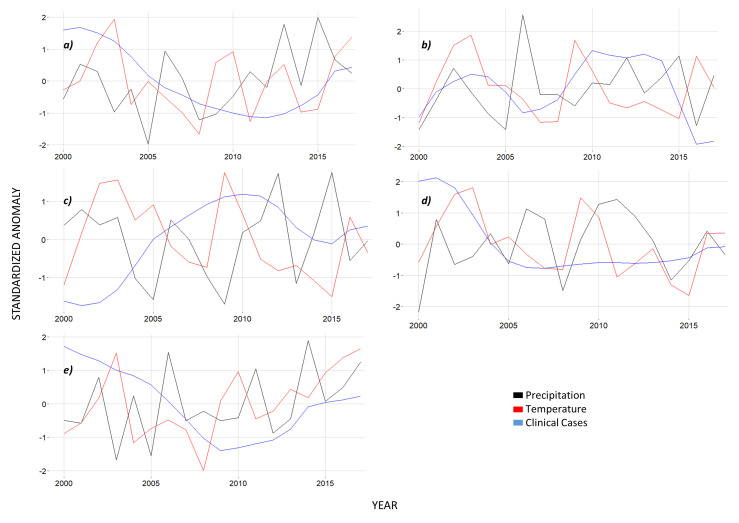
Year-to-year anomalies (standardized) for annual precipitation (black), mean temperature (red), and clinical malaria cases (blue) for Gitega, Burundi ( **a**), Siaya, Kenya ( **b**), Jinja, Uganda ( **c**), Kigali, Rwanda ( **d**), and Morogoro, Tanzania ( **e**), for the period 2000–2017, using CHIRPS data.

Despite heavy investments ( Head *et al.,* 2017) made to combat and eliminate malaria in the study domain, clinical malaria cases showed some correlation with precipitation and temperature. Siaya and Kigali (b and d, respectively, in Figure 2) are good examples where clinical malaria cases corresponded to trends in climate variables, especially the mean temperature. 

Pearson correlation coefficients (PCCs) for five administrative areas recording the highest number of clinical malaria cases per country ( Table 2) showed a positive relationship between temperature and clinical cases, in 22 out of 25 areas under consideration. Burundi recorded the highest positive PCCs (up-to 0.6) between temperature and clinical cases while Uganda recorded the highest negative PCCs (up-to -0.4). Most areas (16 out of 25) recorded a negative correlation between precipitation and clinical malaria cases, with the highest negative correlation being -0.4. The rest showed a marginal positive correlation with the highest being 0.3.

**Table 2.** Pearson correlation coefficients for de-trended precipitation (pr) and mean temperature (tmp) values relative to clinical malaria cases. Values marked with * are significant at 95% significance interval.

	Kenya					Rwanda				
	Busia	Kisumu	Siaya	Kakamega	Bungoma	Kigali	North	South	East	West
pr	-0.01	0.1	0.14	0.19	-0.13	-0.31	-0.3	-0.1	-0.2	-0.1
tmp	0.19	0.23	0.07	0.23	0.4	0.2	0.3	0.4	0.4	0.23
	Tanzania					Burundi				
	Geita	Kagera	Mwanza	Mbeya	Morogoro	Gitega	Kirundo	Muyinga	Ngozi	Ruyigi
pr	0.03	-0.35	-0.18	0.29	0.24	0.24	-0.09	-0.13	0.07	0.07
tmp	0	0.55 *	0.25	0.4	0.44	0.5 *	0.5 *	0.6 *	0.5 *	0.36
	Uganda									
	Iganga	Jinja	Kaabong	Kamuli	Wakiso					
pr	-0.21	-0.33	-0.35	-0.23	-0.21					
tmp	-0.11	0.1	-0.39	-0.03	0.3					

Given that precipitation regimes over the study domain are well-defined (e.g. Nicholson, 2017; Schreck & Semazzi, 2004), the observed negative correlation between precipitation and clinical malaria cases could be as a result of deliberate intensification efforts to combat malaria during the rainy seasons. For instance, an analysis of confirmed malaria cases in Uganda between 2013 and 2016 concluded that the declining cases of Malaria incidence in Uganda was as a result of effective vector-control measures and case management ( Ssempiira *et al.,* 2018). The study showed that a 100% increase in the use of insecticide-treated mosquito nets was associated with a malaria incidence decline of up-to 44% in children below five years of age. In Burundi, where a high correlation between malaria cases and precipitation was recorded, malaria is still a major public health problem responsible for about 25% of all outpatient visits ( USAID, 2016). The relatively high malaria burden could be attributed to, among other factors, limited vector and case control interventions (e.g. Protopopoff *et al.,* 2007; USAID, 2016). Nonetheless, areas that record a positive correlation between rainfall and malaria cases (9 out of 25) imply that more interventions are needed to minimize malaria transmission in the region. The interventions will contribute to the sustenance of gains made and enhance the march towards malaria elimination in East Africa.

### Future changes in precipitation and temperature under 1.5 °C and 2.0 °C GWLs

**Figure 3.**
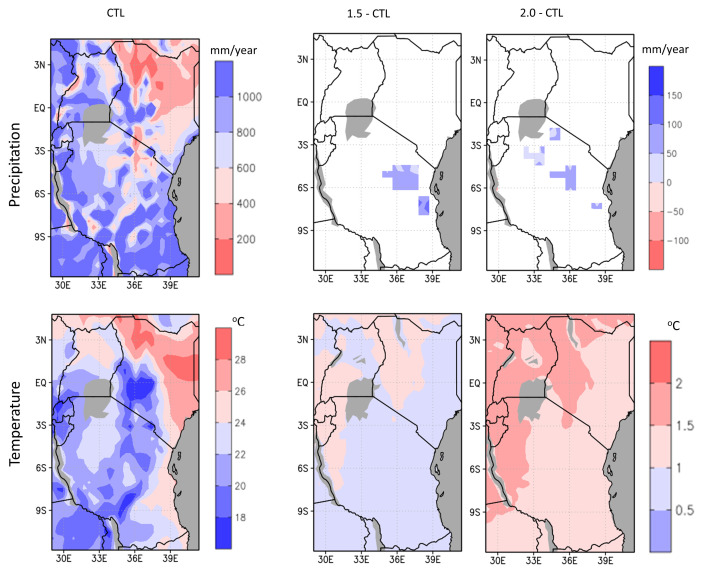
Climatology and future changes (at 95% confidence interval) in precipitation (top row) and temperature (bottom row) under GWL1.5 and GWL2.0 scenarios, relative to the control period (1977–2005). Water bodies are shown in grey.

Most of the study domain (except northern Kenya) recorded an annual precipitation exceeding 400 mm ( Figure 3). Many areas such as the L. Victoria region, most of Tanzania and Uganda, and coastal Kenya showed receipt of annual precipitation exceeding 800 mm. Under 1.5 °C and 2.0 °C GWLs, no significant changes in annual precipitation (at 95% confidence interval) were recorded over the study domain.

While a global warming of up-to 2.0 °C may not necessarily significantly change East Africa’s mean annual precipitation, the region already receives enough precipitation for malaria vector abundance and malaria transmission. Besides, projections show a possibility of increased precipitation intensity and occurrence of extreme events (e.g. Ogega *et al.,* 2020; Weber *et al.,* 2018). More intense and extreme rainfall events in future could enhance the provision of aquatic environments to facilitate more malaria vector abundance and malaria transmission.

In terms of temperature, all areas in the study domain recorded temperatures within the suitability threshold (18–28 °C) for malaria transmission ( Figure 3, bottom row). A few areas such as the Mount Kenya region (around 0.5° S, 36° E) recorded mean annual temperatures below 18° C implying a low likelihood of malaria transmission. Under GWL1.5, temperature changes ranging from 0.5 to 1.5 °C were recorded. This implies a potential increase in the region’s geographical extent for malaria transmission. This is particularly true for many areas in Burundi, Rwanda, and central Kenya (Central and Nairobi provinces) where clinical malaria cases are currently relatively low. A mean temperature increase of between 1–2 °C is expected over the study domain under the GWL2.0. The temperature increase is likely to affect many parts of western Kenya and Tanzania, most of Rwanda, Burundi, and Uganda hence potentially increasing the areas with suitable conditions for malaria transmission. Our results are consistent with findings from similar studies done over the study domain (e.g. Gudoshava *et al*., 2020; Ogega *et al*., 2020; Osima *et al*., 2018).

Global warming is likely to increase the seasons and geographical extents for malaria transmission resulting in more cases and newer malaria hotspots (e.g. Ebi *et al*., 2018; Himeidan & Kweka, 2012; Karungu *et al*., 2019; Peterson, 2009). An analysis of climate projections shows changes in the geographical and seasonal suitability for malaria transmission for East Africa (e.g. Ryan *et al.,* 2020). More investment may be required to facilitate adequate planning and action to minimize the effects of possible future outbreaks. Adequate planning and prioritization of interventions in East Africa is, often, hampered by limitations in data availability (e.g. Deutsch-Feldman *et al.,* 2020; Valle & Lima, 2014). Therefore, more research is needed to enhance the understanding of various factors affecting malaria transmission to inform interventions.

While big investments have been made towards eliminating malaria in East Africa, sustaining the gains made so far remains a big challenge ( Bashir *et al.,* 2019; Nkumama *et al.,* 2017). The current study establishes that, despite the ongoing interventions in East Africa, climatic factors still influence the number of clinical malaria cases. Unless adequate mitigative and adaptive measures are taken, a warming globe is likely to make it difficult to sustain gains made, and slow down the match, towards malaria elimination in East Africa.

## Conclusions

Global warming scenarios of 1.5 °C and 2 °C are likely to increase malaria transmission seasons and geographical extents of malaria transmission in East Africa. Unless interventions are sufficiently intensified, sustaining the gains made towards malaria elimination is likely to be more difficult in a warming climate. Hence, the global community should intensify its collective efforts towards minimizing global warming. Meanwhile, more investment should be made to sustain the gains made and hasten the match towards malaria elimination in East Africa. More research (considering other variables such as altitude, humidity, and vulnerability of communities) is also required to enhance the understanding of spatial and temporal impacts of global warming on malaria transmission in East Africa. Specifically, disease modelling is required to project the new exposed population which will inform future malaria eradication efforts.

## Data availability

### Source data

CORDEX-Africa RCM simulations (files listed in Table 1) were downloaded free of charge from the Deutsches Klimarechenzentrum (DKRZ) accessible at http://bit.ly/2RoIist. To download the data, one needs to create a user account after which data can be downloaded freely for non-commercial use. Gridded mean surface air temperature data (CRU TS v. 4.04) were obtained from the Climatic Research Unit, University of East Anglia and accessed free of charge at https://crudata.uea.ac.uk/cru/data/hrg/. Gridded daily precipitation data (CHIRPS Daily v. 2.0) were obtained from the Climate Hazards Center, University of California, Santa Barbra. The data were freely downloaded from https://bit.ly/3buFCj8. Data on clinical malaria cases for Uganda, Kenya, Burundi, Rwanda, and Tanzania were downloaded (in .csv format) free of charge from the Malaria Atlas Project accessible at https://malariaatlas.org/data-directory/.
