# Supplementary material for: Impact of 1.5 oC and 2 oC global warming scenarios on malaria transmission in East Africa
Source: AAS Open Res. 2021 Mar 15;3:22. Originally published 2020 Jun 3. [Version 3] doi: 10.12688/aasopenres.13074.3 (PMC8008358; doi:10.12688/aasopenres.13074.3)
Supplement: Peer review history

# Peer review: Reviewer response for version 3

**DOI:** 10.21956/aasopenres.14304.r28476

**Published:** 2021-03-26

**Authors:** Masilin Gudoshava

**Affiliations:** 1 National University of Science and Technology, Bulawayo, Zimbabwe

The authors have revised and addressed all the questions raised. The manuscript is in acceptable form to be indexed.

Is the work clearly and accurately presented and does it cite the current literature?

Partly

If applicable, is the statistical analysis and its interpretation appropriate?

Yes

Are all the source data underlying the results available to ensure full reproducibility?

Yes

Is the study design appropriate and is the work technically sound?

Partly

Are the conclusions drawn adequately supported by the results?

Partly

Are sufficient details of methods and analysis provided to allow replication by others?

Partly

Reviewer Expertise:

Climate science

I confirm that I have read this submission and believe that I have an appropriate level of expertise to confirm that it is of an acceptable scientific standard.

# Peer review: Reviewer response for version 2

**DOI:** 10.21956/aasopenres.14263.r28098

**Published:** 2020-12-14

**Authors:** Ramesh C. Dhiman

**Affiliations:** 1 ICMR-National Institute of Malaria Research, New Delhi, Delhi, India

The authors have revised the paper in light of the suggestions of the reviewers and it is acceptable for indexing.

Is the work clearly and accurately presented and does it cite the current literature?

Yes

If applicable, is the statistical analysis and its interpretation appropriate?

Yes

Are all the source data underlying the results available to ensure full reproducibility?

Yes

Is the study design appropriate and is the work technically sound?

No

Are the conclusions drawn adequately supported by the results?

No

Are sufficient details of methods and analysis provided to allow replication by others?

Yes

Reviewer Expertise:

Epidemiology and vector control of vector borne diseases. Climate change impacts on VBDs. Early warning of outbreaks of malaria and dengue using climate and satellite data.

I confirm that I have read this submission and believe that I have an appropriate level of expertise to confirm that it is of an acceptable scientific standard.

# Peer review: Reviewer response for version 2

**DOI:** 10.21956/aasopenres.14263.r28097

**Published:** 2020-11-12

**Authors:** Masilin Gudoshava

**Affiliations:** 1 National University of Science and Technology, Bulawayo, Zimbabwe

Introduction section: Uganda, for instance, tops East Africa “hereinafter East Africa” seems redundant in this case, since its just a repetition.

 The authors only use 4 realizations of the RCMS however one of the justification of use of RCP8.5, is that the scenario provides the largest number of ensemble members, I would suggest that they drop this justification as it doesn’t seem to fit with the design of the analysis.

 I am still finding it hard to understand how the authors calculated/identified the mid year for the different global warming levels using the 2 GCMs. Is this calculation from Nikulin *et al.*, 2018 using all the GCMs? A brief explanation on how this is done will be helpful. 

 A recommendation is drawn about Malaria cases and precipitation however these values are not statistically significant. I suggest that the authors mostly focus on the temperature which has statistical significant correlations. (“record a positive correlation between rainfall and malaria cases (9 out of 25) imply that more interventions are needed to minimize malaria transmission in the region.”)

Is the work clearly and accurately presented and does it cite the current literature?

Partly

If applicable, is the statistical analysis and its interpretation appropriate?

Yes

Are all the source data underlying the results available to ensure full reproducibility?

Yes

Is the study design appropriate and is the work technically sound?

Partly

Are the conclusions drawn adequately supported by the results?

Partly

Are sufficient details of methods and analysis provided to allow replication by others?

Partly

Reviewer Expertise:

climate science

I confirm that I have read this submission and believe that I have an appropriate level of expertise to confirm that it is of an acceptable scientific standard, however I have significant reservations, as outlined above.

# Response

**Published:** 2020-12-26

**Authors:** Obed Matundura Ogega

**Affiliations:** The African Academy of Sciences, Kenya

**Comment: **Introduction section: Uganda, for instance, tops East Africa “hereinafter East Africa” seems redundant in this case, since its just a repetition.

***Response: **Now revised to exclude “hereinafter East Africa”*

**Comment: **The authors only use 4 realizations of the RCMS however one of the justification of use of RCP8.5, is that the scenario provides the largest number of ensemble members, I would suggest that they drop this justification as it doesn’t seem to fit with the design of the analysis.

***Response: **now revised to exclude “provides the largest set of ensemble members than other RCPs and”*

**Comment:** I am still finding it hard to understand how the authors calculated/identified the mid year for the different global warming levels using the 2 GCMs. Is this calculation from Nikulin et al., 2018 using all the GCMs? A brief explanation on how this is done will be helpful.

***Response: **This part has now been revised for coherence in paragraph 3 of the Data Analysis section.*

**Comment: **A recommendation is drawn about Malaria cases and precipitation however these values are not statistically significant. I suggest that the authors mostly focus on the temperature which has statistical significant correlations. (“record a positive correlation between rainfall and malaria cases (9 out of 25) imply that more interventions are needed to minimize malaria transmission in the region.”)

***Response: **this is well-noted. As we focus on temperature, we also want the region to keep monitoring the contribution of precipitation on malaria transmission. While our results show minimal effect of precipitation on malaria cases, the region needs to hasten the interventions to avoid reversing the gains made towards malaria elimination – hence the recommendation.*

# Peer review: Reviewer response for version 1

**DOI:** 10.21956/aasopenres.14170.r27822

**Published:** 2020-09-23

**Authors:** Ramesh C. Dhiman

**Affiliations:** 1 ICMR-National Institute of Malaria Research, New Delhi, Delhi, India

- The study has used 20-25 ^o^C as thresholds of temperature within which maximum suitability for survival of Anopheles mosquitoes is achieved. What is the basis of selecting this range? This requires clarification. Further, instead of selecting the range for survival of mosquitoes, the threshold for transmission of malaria (sporogony) would have been ideal as the present communication deals with future scenario of malaria and not the anopheles vector. Mosquitoes are known to survive even at lower than 13 ^o^C temperature also (IPCC, 2001). The temperature between 18-32 ^o^C is considered suitable for transmission of malaria (Craig *et al.*, 1999 ^1^). Further Mordecai *et al.* (2013 ^2^) found that the upper threshold is 28 ^o^C, bit lower than 32 ^o^C.
- In the study, the threshold of rainfall has been taken of Aedes mosquitoes. Anopheles and Aedes mosquitoes are quite different in their ecology, biology and climatic requirements. Therefore, using the thresholds of rainfall meant for Aedes, does not seem appropriate for Anopheles.
- The authors are advised to consult Craig *et al. *(1999 ^1^) on a climate-based distribution model of malaria in SS Africa where 80 mm rainfall has been considered as the threshold of rainfall causing outbreaks of malaria.
- Using only RCP 8.5 of climate model does not seem appropriate; RCP 4.5 being moderate, would have been ideal.
- Relevance of Fig 2 is not clear.
- Fig 3: Analysis with annual precipitation data is not desirable for a climate-sensitive disease like malaria. The significance of depicting water bodies is not clear.
- Page 6, last paragraph: ‘In terms of temperature, all areas in the study domain record temperatures within suitability threshold (13-30 ^o^C) for malaria vectors’ is not appropriate as at lower than 18 ^o^C temperature, transmission is not possible as the mosquitoes would require 56 days to complete sporogony (Craig *et al.*, 1999 ^1^).
- The authors have undertaken painstaking efforts in analyzing the projected scenario of malaria in view of projected rise in temperature. The objective of the study is also very pertinent. But owing to methodological issues stated above, reanalysis of data is required.

Is the work clearly and accurately presented and does it cite the current literature?

Yes

If applicable, is the statistical analysis and its interpretation appropriate?

Yes

Are all the source data underlying the results available to ensure full reproducibility?

Yes

Is the study design appropriate and is the work technically sound?

No

Are the conclusions drawn adequately supported by the results?

No

Are sufficient details of methods and analysis provided to allow replication by others?

Yes

Reviewer Expertise:

Epidemiology and vector control of vector borne diseases. Climate change impacts on VBDs. Early warning of outbreaks of malaria and dengue using climate and satellite data.

I confirm that I have read this submission and believe that I have an appropriate level of expertise to confirm that it is of an acceptable scientific standard, however I have significant reservations, as outlined above.

## References

[1] : A Climate-based Distribution Model of Malaria Transmission in Sub-Saharan Africa. *Parasitology Today*.1999;15(3) : 10.1016/S0169-4758(99)01396-4 105-111 10.1016/S0169-4758(99)01396-4 10322323

[2] : Optimal temperature for malaria transmission is dramatically lower than previously predicted. *Ecol Lett*.2013;16(1) : 10.1111/ele.12015 22-30 10.1111/ele.12015 23050931

# Response

**Published:** 2020-10-17

**Authors:** Obed Matundura Ogega

**Affiliations:** The African Academy of Sciences, Kenya

The authors are very grateful for the Reviewers’ comments. The comments and suggestions have been very useful during the revision process and have been incorporated into the revised manuscript. We believe the quality of the revised manuscript has been improved significantly. The specific actions in response to the reviewers’ comments are outlined as follows.

**Reviewer #2:**

Comment: The study has used 20-250 C as thresholds of temperature within which maximum suitability for survival of Anopheles mosquitoes is achieved. What is the basis of selecting this range? This requires clarification. Further, instead of selecting the range for survival of mosquitoes, the threshold for transmission of malaria (sporogony) would have been ideal as the present communication deals with future scenario of malaria and not the anopheles vector. Mosquitoes are known to survive even at lower than 130 C temperature also (IPCC, 2001). The temperature between 18-320C is considered suitable for transmission of malaria (Craig et al., 19991). Further Mordecai et al. (20132) found that the upper threshold is 280 C, bit lower than 320 C.

*Response: This is well-noted. The 20-25 ^o^C is the range within which the mosquito abundance increases to maximum. However, we have revised our manuscript to focus on malaria transmission and not mosquito survival (see lines 143 – 146).*

Comment: In the study, the threshold of rainfall has been taken of Aedes mosquitoes. Anopheles and Aedes mosquitoes are quite different in their ecology, biology and climatic requirements. Therefore, using the thresholds of rainfall meant for Aedes, does not seem appropriate for Anopheles.

*Response: We have revised the section on rainfall to focus on malaria transmission rather than Aedes mosquitoes (see lines 136-146).*

Comment: The authors are advised to consult Craig et al. (19991) on a climate-based distribution model of malaria in SS Africa where 80 mm rainfall has been considered as the threshold of rainfall causing outbreaks of malaria.

*Response: This has been revised. We now discuss precipitation patterns in general without referring to specific thresholds (e.g. lines 136-143).*

Comment: Using only RCP 8.5 of climate model does not seem appropriate; RCP 4.5 being moderate, would have been ideal.

*Response: Considering the current global greenhouse emission scenarios, RCP 8.5 gives a more realistic representation of global warming scenario compared to the RCP 4.5. Additionally, more simulations are available for RCP 8.5 compared to RCP 4.5. This explanation has now been given in lines 40-44.*

Comment: Relevance of Fig 2 is not clear.

*Response: Fig. 2 has been discarded and its details included in the narrative (see lines 127-131).*

Comment: Fig 3: Analysis with annual precipitation data is not desirable for a climate-sensitive disease like malaria. The significance of depicting water bodies is not clear.

*Response: Fig. 3 has been revised to exclude the spatial plots. We computed and compared year-to-year standardized anomalies for precipitation, temperature, and clinical malaria cases to show a general relationship in trend behaviour for the three variables. Standardized anomalies free data from location and distribution influences hence giving a more realistic trend. While malaria prevention efforts are designed to counter the potential effect of weather and climate in malaria transmission, we wanted to show if any discernible relationships still exist between climatic factors (malaria and temperature) and annual clinical cases. We showed major water bodies (Lake Victoria and Indian Ocean) in grey to focus the analysis only on land.*

Comment: Page 6, last paragraph: ‘In terms of temperature, all areas in the study domain record temperatures within suitability threshold (13-300C) for malaria vectors’ is not appropriate as at lower than 18 0C temperature, transmission is not possible as the mosquitoes would require 56 days to complete sporogony (Craig et al., 19991).

*Response: The manuscript has been revised accordingly (e.g. lines 137-141).*

Comment: The authors have undertaken painstaking efforts in analyzing the projected scenario of malaria in view of projected rise in temperature. The objective of the study is also very pertinent. But owing to methodological issues stated above, reanalysis of data is required.

*Response: We appreciate your comments and have made the suggested revisions to make the current version of the manuscript more plausible.*

Comment: Additional references

1. Craig M, Snow R, le Sueur D: A Climate-based Distribution Model of Malaria Transmission in Sub-Saharan Africa. Parasitology Today. 1999; 15 (3): 105-111 Publisher Full Text

2. Mordecai EA, Paaijmans KP, Johnson LR, Balzer C, et al.: Optimal temperature for malaria transmission is dramatically lower than previously predicted.Ecol Lett. 2013; 16 (1): 22-30 PubMed Abstract | Publisher Full Text

*Response: We are grateful for these suggestions – which have now been used to enrich the manuscript.*

# Peer review: Reviewer response for version 1

**DOI:** 10.21956/aasopenres.14170.r27675

**Published:** 2020-08-10

**Authors:** Masilin Gudoshava

**Affiliations:** 1 National University of Science and Technology, Bulawayo, Zimbabwe

- In the introduction section paragraph 2, the authors discuss that there have been some studies done towards understanding the impact of climate over the region at the different GWL and state that there are no conclusive literature on the impact. Did this statement mean to say no conclusive literature on impacts in sectors such as health, agriculture water etc?  
- Methods Climate Modelling paragraph 1: A statement explaining why the authors only chose RCP85 scenario is required in this case as the different scenarios can have differing impacts.
- Table 1 indicates that data was downloaded from 2071-2100 however this is not the same time period that was analyzed. This is a typo that needs to be corrected.  
- In paragraph 2 of the data analysis section the authors state that 2022 and 2037 have been identified as mid-years for 30-year windows when GWL1.5 and GWL2.0, respectively, are likely to be first experienced. However different GCMs hit these levels at different times, is it the assumption in this manuscript that both these GCMs will reach the GWL at the same time? I would suggest that the authors rework on this and use the GWL for the different GCMS.
- The malaria temperature survival has two different threshold values (13-30 ^o^ C) in one section and in another section it is written as 15-30 ^o^ C. Double check this. 
- Trends in temperature, precipitation, and clinical malaria cases in E. Africa paragraph graph two: an explanation on why malaria clinical cases and temperature are negatively correlated over Uganda is needed here, since in all the other countries the correlation is positive. 
- Trends in temperature, precipitation, and clinical malaria cases in E. Africa paragraph three: could the negative correlations be caused by the washing away of the eggs due to high rainfall rather than intensification of efforts to combat malaria?
- Figure 3: The line plots do not show any obvious relationships between the malaria clinical cases and the temperature/rainfall - could this be because people are taking preventive measures? Is it possible to obtain the actual vector data and do the analysis using this rather than the clinical cases? 
- Figure 4: The caption seems incorrect, I would not expect rainfall of up to 700mm/day in any season over East Africa, also in the write-up it is written mm/year. 
- A discussion on the large scale drivers and malaria cases could be helpful in explaining any likely changes in the future of the reported clinical cases. 

Is the work clearly and accurately presented and does it cite the current literature?

Partly

If applicable, is the statistical analysis and its interpretation appropriate?

Yes

Are all the source data underlying the results available to ensure full reproducibility?

Yes

Is the study design appropriate and is the work technically sound?

Partly

Are the conclusions drawn adequately supported by the results?

Partly

Are sufficient details of methods and analysis provided to allow replication by others?

Partly

Reviewer Expertise:

climate science

I confirm that I have read this submission and believe that I have an appropriate level of expertise to confirm that it is of an acceptable scientific standard, however I have significant reservations, as outlined above.

# Response

**Published:** 2020-10-17

**Authors:** Obed Matundura Ogega

**Affiliations:** The African Academy of Sciences, Kenya

The authors are very grateful for the Reviewers’ comments. The comments and suggestions have been very useful during the revision process and have been incorporated into the revised manuscript. We believe the quality of the revised manuscript has been improved significantly. The specific actions in response to the reviewers’ comments are outlined as follows.

**Reviewer #1**:

Comment: In the introduction section paragraph 2, the authors discuss that there have been some studies done towards understanding the impact of climate over the region at the different GWL and state that there are no conclusive literature on the impact. Did this statement mean to say no conclusive literature on impacts in sectors such as health, agriculture water etc.? 

*Response: Indeed, we meant to say impacts on the health sector. This has been corrected (see line 22).*

Comment: Methods Climate Modelling paragraph 1: A statement explaining why the authors only chose RCP85 scenario is required in this case as the different scenarios can have differing impacts.

*Response: An explanation has now been given in lines 40-44.*

Comment: Table 1 indicates that data was downloaded from 2071-2100 however this is not the same time period that was analyzed. This is a typo that needs to be corrected. 

*Response: Now revised to show 2008-2052 (see line 51).*

Comment: In paragraph 2 of the data analysis section the authors state that 2022 and 2037 have been identified as mid-years for 30-year windows when GWL1.5 and GWL2.0, respectively, are likely to be first experienced. However different GCMs hit these levels at different times, is it the assumption in this manuscript that both these GCMs will reach the GWL at the same time? I would suggest that the authors rework on this and use the GWL for the different GCMS.

*Response: It is indeed true that different GCMs hit the said levels at different times. Years 2022 and 2037 represent values for an ensemble mean of the subset of GCM simulations used in CORDEX. Our analysis used an ensemble mean of these CORDEX GCM simulations. The section has been revised for coherence (see lines 86-97).*

Comment: The malaria temperature survival has two different threshold values (13-30 o C) in one section and in another section it is written as 15-30 o C. Double check this.

*Response: This has been revised (in both cases) to 18 – 28 ^o^C (see e.g. lines 132-134).*

Comment: Trends in temperature, precipitation, and clinical malaria cases in E. Africa paragraph graph two: an explanation on why malaria clinical cases and temperature are negatively correlated over Uganda is needed here, since in all the other countries the correlation is positive.

*Response: We have provided more information in lines 172-184.*

Comment: Trends in temperature, precipitation, and clinical malaria cases in E. Africa paragraph three: could the negative correlations be caused by the washing away of the eggs due to high rainfall rather than intensification of efforts to combat malaria?

*Response: Egg flushing can indeed be a possibility. However, no major rainfall changes have been recorded during the period under study. Rainfall has mostly remained within the normal range (as shown in Figure 2). As detailed in lines 172-184 of the revised manuscript, the relatively low/high correlation between malaria cases and climatic factors in Uganda/Burundi result from case and vector control investment.*

Comment: Figure 3: The line plots do not show any obvious relationships between the malaria clinical cases and the temperature/rainfall - could this be because people are taking preventive measures? Is it possible to obtain the actual vector data and do the analysis using this rather than the clinical cases?

*Response: Please refer to our response in 7 above. The use of actual vector data is out of the scope of the current study. We have recommended a further study (see lines 231-235) where such data can be obtained and used.*

Comment: Figure 4: The caption seems incorrect, I would not expect rainfall of up to 700mm/day in any season over East Africa, also in the write-up it is written mm/year.

*Response: This has been corrected (see Figure 3).*

Comment: A discussion on the large scale drivers and malaria cases could be helpful in explaining any likely changes in the future of the reported clinical cases.

*Response: Thank you for this suggestion. We have provided more information in lines 218-233.*
